# Association between Gut Microbiota and Breast Cancer: Diet as a Potential Modulating Factor

**DOI:** 10.3390/nu15214628

**Published:** 2023-10-31

**Authors:** Duygu Altinok Dindar, Brie Chun, Amy Palma, John Cheney, Madeline Krieger, Kristin Kasschau, Keaton Stagaman, Zahi I. Mitri, Shaun M. Goodyear, Jackilen Shannon, Lisa Karstens, Thomas Sharpton, Zhenzhen Zhang

**Affiliations:** 1 Cancer Early Detection Advanced Research Center, Knight Cancer Institute, Oregon Health & Science University, Portland, OR 97239, USA; altinokd@ohsu.edu (D.A.D.); john.h.cheney@gmail.com (J.C.); kriegema@ohsu.edu (M.K.); shannoja@ohsu.edu (J.S.); 2 Division of Hematology & Oncology, Oregon Health & Science University, Portland, OR 97239, USAgoodyear@ohsu.edu (S.M.G.); 3 Division of Oncological Sciences, Oregon Health & Science University, Portland, OR 97239, USA; palmaa@ohsu.edu; 4 Department of Microbiology, Department of Statistics, Center for Quantitative Life Sciences, Oregon State University, Corvallis, OR 97331, USA; kristin.kasschau@oregonstate.edu (K.K.); kstagaman@gmail.com (K.S.); thomas.sharpton@oregonstate.edu (T.S.); 5 British Columbia Cancer, Vancouver, BC V5Z 4E6, Canada; zahi.mitri@bccancer.bc.ca; 6 Department of Medical Informatics and Clinical Epidemiology, Oregon Health & Science University, Portland, OR 97239, USA; karstens@ohsu.edu; 7 Department of Obstetrics and Gynecology, Oregon Health and Science University, Portland, OR 97239, USA

**Keywords:** gut microbiome, breast cancer, Acidaminococus, Hungatella, Tyzzerella

## Abstract

Breast cancer (BCa) has many well-known risk factors, including age, genetics, lifestyle, and diet; however, the influence of the gut microbiome on BCa remains an emerging area of investigation. This study explores the connection between the gut microbiome, dietary habits, and BCa risk. We enrolled newly diagnosed BCa patients and age-matched cancer-free controls in a case-control study. Comprehensive patient data was collected, including dietary habits assessed through the National Cancer Institute Diet History Questionnaire (DHQ). 16S rRNA amplicon sequencing was used to analyze gut microbiome composition and assess alpha and beta diversity. Microbiome analysis revealed differences in the gut microbiome composition between cases and controls, with reduced microbial diversity in BCa patients. The abundance of three specific microbial genera—Acidaminococus, Tyzzerella, and Hungatella—was enriched in the fecal samples taken from BCa patients. These genera were associated with distinct dietary patterns, revealing significant associations between the presence of these genera in the microbiome and specific HEI2015 components, such as vegetables and dairy for Hungatella, and whole fruits for Acidaminococus. Demographic characteristics were well-balanced between groups, with a significantly higher body mass index and lower physical activity observed in cases, underscoring the role of weight management in BCa risk. Associations between significant microbial genera identified from BCa cases and dietary intakes were identified, which highlights the potential of the gut microbiome as a source of biomarkers for BCa risk assessment. This study calls attention to the complex interplay between the gut microbiome, lifestyle factors including diet, and BCa risk.

## 1. Introduction

Breast cancer (BCa) is the leading type of cancer among women worldwide, accounting for approximately 25% of all incident cancers in women [1]. In 2020, there were 2,261,419 new cases of invasive BCa and 684,996 deaths [2]. While the risk factors for BCa are multifactorial (e.g., age, race, genetics, lifestyle, diet, and microenvironment), the underlying causes are not yet fully understood [3].

Several epidemiological and nutritional studies have identified potential risk factors for BCa, such as a high-fat diet [4], red and processed meat consumption [5], lack of physical activity [6], alcohol consumption [7], and tobacco use [8]. However, the mechanisms by which these factors contribute to BCa development remains elusive. Furthermore, genetic risks only account for a small percentage (~10%) of cases [9]. Therefore, exploring potential risk factors beyond genetics is crucial to advancing our understanding of BCa formation.

Emerging evidence suggests that the gut microbiome may be involved in BCa tumorigenesis and progression [10]. The gut microbiome is a diverse community of microorganisms, including bacteria, viruses, and fungi, that reside in the gut and are involved in regulating various physiological processes [11]. Imbalances in gut microbial populations are associated with an increased risk of BCa [12,13], and specific compounds produced by gut bacteria may promote or inhibit BCa development [14], as well as influence the immune response to abnormal cells [15,16]. Thus, the gut microbiome may serve as a rich source of potential biomarkers that can inform patient prognosis and response to therapy [17,18,19].

Diet plays a significant role in shaping the microbiome, and in turn, the intestinal microbiome can subsequently play an important role in modulating the risk of several chronic diseases, including cancer [20]. Importantly, the gut microbiome can influence the relationship between dietary intake, inflammation, and cancer risk [21]. Since the gut microbiome is influenced by a variety of factors (e.g., diet, lifestyle, and genetics), it is important to consider these factors when studying the gut microbiome in BCa patients. In this pilot study, we investigated the association between the gut microbiome, diet, and BCa, and sought to identify specific microbial taxa that may pose a higher risk for the development of the disease.

## 2. Methods

### 2.1. Participants and Sample Collection

Between March 2020 to October 2021, we enrolled 42 cases of newly diagnosed, treatment-naïve female BCa patients at Oregon Health & Science University (OHSU) and 44 age-matched cancer-free controls identified from an OHSU volunteer registry. Inclusion and exclusion criteria were established to ensure the selection of appropriate participants for our research. For breast cancer participants, inclusion criteria required individuals to be female, aged between 20 and 89 years, proficient in English, and to possess a biopsy-confirmed diagnosis of breast cancer prior to initiating any treatment, including surgery, chemotherapy, or radiation therapy. Non-cancer control participants were also limited to females aged 20 to 89 who were English speakers. Additionally, individuals aged 45 to 89 were required to have had a routine mammogram with non-suspicious results within the past 2 years. Exclusion criteria encompassed individuals with a history of prior cancer (excluding non-melanoma skin cancer), those unable to provide legal consent, and those either under 20 or above 89 years of age. For control participants, the exclusion criteria also involved specific medical conditions and recent surgical history, including inflammatory bowel disease, diverticulitis, gastric banding, bypass surgery, or recent gastric or intestinal surgery. All participants were required to provide signed informed consent. The study collected comprehensive epidemiological data including demographics, lifestyle, physical activity, diet questionnaires, and fecal samples. Fecal samples were collected using OMNIgene^®^. gut kit, aliquoted into cryovials, and stored at −80 °C. This study was approved by OHSU’s Institutional Review Board (IRB) (IRB#: 000020449).

### 2.2. Diet and Healthy Eating Index Assessment

The dietary intake of participants was assessed using the National Cancer Institute (NCI) Diet History Questionnaire (DHQ), a validated web-based tool commonly employed in large-scale research studies [22]. This questionnaire gathers comprehensive information on food and nutrient intake, enabling the exploration of relationships between dietary habits, nutrient intake, microbiome diversity, and the risk of cancer.

The DHQ provides food items and groups necessary for the calculation of the Healthy Eating Index-2015 (HEI-2015), which is a scoring system used to evaluate overall diet quality based on adherence to the 2015–2020 Dietary Guidelines for Americans [23]. The HEI-2015 assesses the intake of various food components and assigns scores to different aspects of the diet, including the following 13 components: total fruits, whole fruits, total vegetables, greens and beans, total protein foods, seafood and plant proteins, whole grains, dairy, fatty acids, refined grains, sodium, added sugars, and saturated fats.

The ‘saturated fats’ and ‘added sugars’ components were converted into percentages of total energy intake, while the other food components were adjusted to amounts per 1000 kcal, except for fatty acids. The first six components were assigned a maximum of 5 points each, while the remaining seven components had a maximum assignment of 10 points each.

A higher intake of whole grains, dairy, and fatty acids received 10 points, while a higher intake of total fruits, whole fruits, total vegetables, greens and beans, total protein foods, seafood, and plant proteins received 5 points. Conversely, a lower intake of these items scored 0. A higher intake of refined grains, sodium, added sugars, and saturated fats received a 0 score, while a lower intake of these items received 10 points. Consequently, the HEI-2015 provided an overall score ranging from 0 to 100, with a higher score indicating better adherence to the recommended dietary guidelines and reflecting a healthier diet quality [23].

Evaluation of physical activities was assessed using three levels of physical activities: light, moderate, and strenuous. Participants were asked to report the duration of each of these three levels per week (<1 h, 1-<2 h, 2-<3 h, 3-<5 h, and 5 or more hours). We also calculated metabolic equivalent of task (MET) scores for each level and summed the MET scores for each participant.

Furthermore, we inquired about participants’ feelings by asking questions such as “About how much of the time would you say you felt downhearted and blue” across different lifetimes, that is, (1) in the past year, (2) around the ages of 15 and (3) 25 years old. This question is modified from Short-Form Health Survey-12 (SF-12)’s related question. SF-12 is one of the most widely used instruments for assessing self-reported health-related quality of life [24].

### 2.3. Microbiome Data

#### 2.3.1. Bacterial DNA Extraction and Next-Generation Sequencing

Aliquots of fecal samples were transferred into 96-well plates for DNA extraction using the QIAGEN^®^ QIAamp PowerFecal Pro^®^ DNA extraction kit. The samples were lysed mechanically using a QIAGEN^®^ TissueLyser II. DNA was extracted from fecal samples using the QIAGEN DNeasy R PowerSoil RNA DNA isolation kit (QIAGEN, Germantown, MD USA) as per manufacturer instructions, with the exception of an additional heat incubation of 10 min at 65 °C immediately prior to bead beating. The 16S rRNA gene was amplified from the extracted DNA with PCR and primers designed to target the V4 region [25]. Subsequent amplicons were quantified using the Qubit R HS kit (Thermo Fisher, Waltham, MA, USA), then pooled and cleaned using the UltraClean R PCR clean-up kit (MO BIO, Carlsbad, CA, USA). Cleaned amplicons were sequenced at the Center for Quantitative Life Sciences at Oregon State University using the Illumina MiSeq platform, employing the 2 × 300 sequencing reaction. The average sequencing depth per sample was 130,000 reads (Illumina, Inc., San Diego, CA, USA).

#### 2.3.2. Bioinformatics and Statistical Analysis

Raw sequences were processed using the dada2 R package [26]. The analysis of microbiota profiles involved quality filtering, merging reads, and assigning amplicon sequence variants (ASVs) from the raw sequences, which were then used to estimate taxon abundances. Taxonomy was assigned to each ASV at the genus level using the Silva 16S database [27]. The alignment of ASV sequences was performed using guide sequences from the Silva 16S database. The aligned sequences were used to construct a phylogenetic tree using FastTree [28] to estimate an approximate maximum likelihood phylogeny of the ASVs. ASVs that were either not assigned at the kingdom taxonomic level or that were assigned as Chloroplast or Mitochondria at the order or family taxonomic levels were excluded from the analysis. The phyloseq [29] R package was used to rarify samples and quantify α- (richness and evenness) and β-diversity (e.g., Bray-Curtis) distances [30].

Alpha diversity measures were used to assess the diversity of species or taxa within a single microbial community or sample. The alpha-diversity indices (Shannon, Inverse Simpson, Observed, and Pielou’s Evenness) were analyzed using the ANOVA and Wilcoxon rank-sum test [31,32,33,34]. A combination of these measures obtains a more complete picture of microbial diversity in both groups.

Beta diversity analysis, as measured by the Jaccard distance and Bray-Curtis dissimilarity metric, examined the compositional differences in fecal microbiota between the two groups and each serves a specific purpose in assessing the compositional dissimilarity between microbial communities [35]. In particular, the Jaccard distance measures the presence or absence of species (or taxa) in two different microbial communities, and as a result, can be more sensitive to rare taxa as compared to metrics that weight beta-diversity by taxon abundance [35,36]. Comparing results between analyses using these two metrics can reveal cases wherein rare taxa are important to a biological phenomenon [35]. The statistical significance of beta-diversity was measured using permutational multivariate analysis of variance (PERMANOVA).

Linear Discriminant Analysis Effect Size (LEFSe) is a statistical method commonly used in microbiome studies to identify microbial taxa (e.g., bacteria, archaea) or functional features (e.g., genes, pathways) that are differentially abundant between two or more biological groups, such as healthy individuals versus those with a particular disease [37]. LEfSe aims to find features that statistically explain differences between groups and quantify discrimination scores that represent each feature’s effect size. LEfSe combines non-parametric statistical tests (Kruskal-Wallis and Wilcoxon rank-sum tests) with linear discriminant analysis (LDA). The Kruskal-Wallis test is used to identify taxa or features that show significant differences in abundance between groups. The Wilcoxon rank-sum test is applied to assess whether differences are statistically significant. Taxonomic levels with LDA values higher than 2 at a *p*-value < 0.05 were considered statistically significant. The ggplot2 package in the R program (version 3.4.3) was used to visualize the LEfSe differences between the groups.

Demographic and lifestyle characteristics were compared between groups using *t*-tests for continuous variables and chi-squared tests for categorical variables. Associations between dietary factors and identified microbiome genera were analyzed using either a parametric two-sample *t*-test or a non-parametric Wilcoxon-Mann-Whitney two-sample test (depending on the distribution of the data). Normally distributed variables were analyzed using the Shapiro-Wilk test. The dietary-related analyses were conducted using SAS software version 9.4 (SAS Institute, Cary, NC, USA).

## 3. Results

### 3.1. Participant Characteristics

A total of n = 42 breast cancer cases and n = 44 cancer-free controls who donated their stool samples were included for microbiome analyses, and n = 39 breast cancer cases and n = 44 controls who had completed the DHQ questionnaire were included for diet-related analyses (Supplemental Figure S1). Both the case and control groups were predominantly non-Hispanic white, with a mean age of 60.3 years (SD = 11.3, Range = 38–80) and a mean BMI of 28 (SD = 6.2) (Table 1). No significant differences were found in other demographic factors between the case and control groups, including: age, race, family history of BCa, parity status, menopausal status, and menarche age. Among patients with BCa, 32 (76%) were post/peri-menopausal, 27 participants (64%) had no history of cancer, and 12 participants (28.6%) were nulliparous. BMI was higher among cases (BMI = 28.1 kg/m^2^, SD = 6.2) compared to controls (25.1 kg/m^2^, SD = 5.6, *p* = 0.02) (Table 1).

Among BCa cases, 7 were ductal carcinoma in situ (DCIS) and 35 were invasive (American Joint Committee on Cancer, Collaborative Staging Version 2.04). Thirty-six tumors were estrogen receptor (ER)-positive, 30 were progesterone receptor (PR)–positive, and 3 were positive for human epidermal growth factor receptor 2 (HER2). We next examined various lifestyle factors, including: dietary habits, marital status, employment status, tobacco and alcohol consumption, sleep quality scores, hormonal therapy history, probiotic use, and physical activity. Except for physical activity, no significant differences were observed in these lifestyle factors between the case and control groups. Statistically significant differences were found between cases and controls in the levels of total energy expenditure from recreational physical activity measured in MET-minutes/week (*p* = 0.02). Additionally, compared to controls, cases reported higher levels of emotional stress in the past year (*p* = 0.02) and by their recollection, their level of stress at the age of 25 (*p* = 0.01) (Table 2).

### 3.2. Microbiome Composition

We observed differences in both the alpha- and beta-diversity of the gut microbiome as a function of BCa. First, the alpha diversity of the BMI-adjusted BCa group was significantly lower than the controls using the Shannon index (*p* = 0.012), Observed (*p* = 0.025), Inverse Simpson (*p* = 0.005), and Pielou (*p* = 0.028) alpha diversity measures (Table 3). Second, while significant differences were not observed between groups when using the Bray-Curtis dissimilarity measure (*p* = 0.23), the beta-diversity did significantly differ between the two groups when using the Jaccard distance (*p* = 0.04) (Table 4). We also did not observe beta-diversity differences by breast cancer subtype or staging.

**Figure 1:**
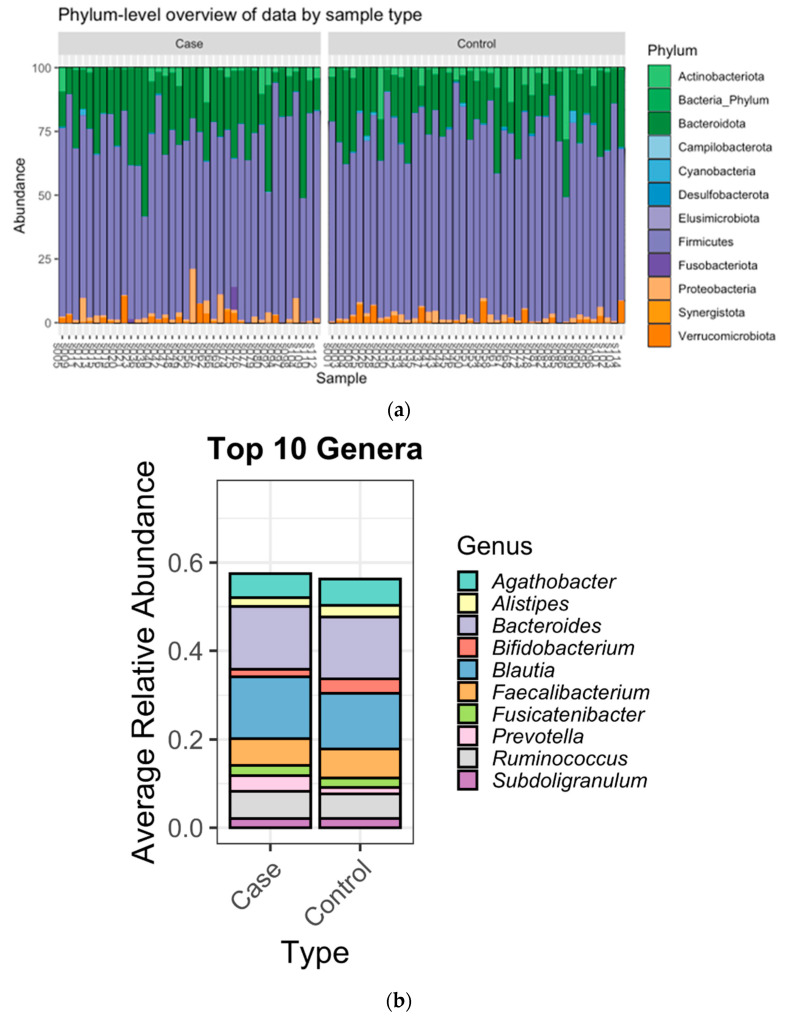
(**a**): Bar plots of the gut microbiota taxonomic profiles at the phylum level. (**b**): Top ten genus-level abundance stratified by case/control status. Relative abundance of taxonomic profile of BC and HC groups at phylum and genus levels.

**Figure 2:**
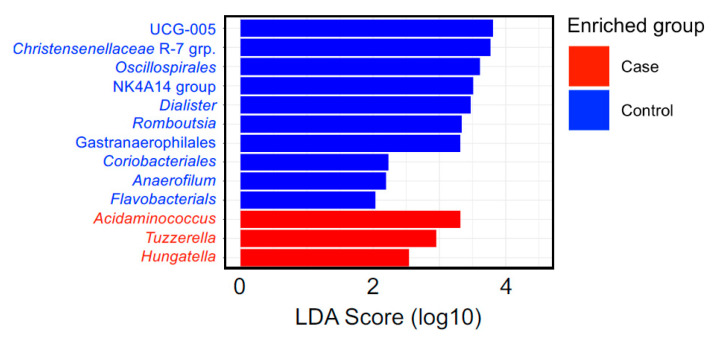
Linear discriminant analysis effect size (LEfSe) analysis of fecal microbiome at genus level of the participants in the control and BCa group samples. An LDA (score > 2) effect size-based bar graph showed bacterial genus enriched in feces from cancer-free controls and BCa groups.

**Figure 3:**
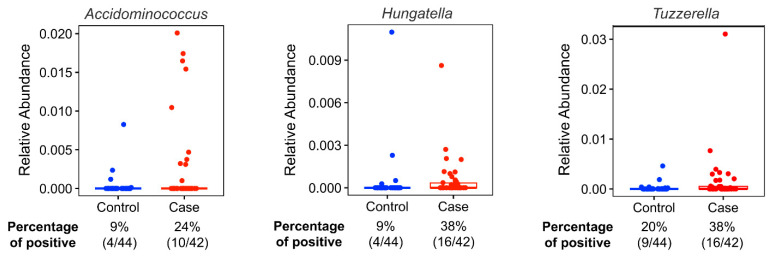
Relative abundances (%) of identified biomarker candidates’ taxonomic abundances by case-control status. Twenty-four percent of the cases (10/42) and 9% of the controls (4/44) have Acidaminococus in their microbiome. The relative abundances (%) of the participants who have Acidaminococus species in their gut microbiome ranges between 1.850% and 0.010%. Thirty-eight percent of the cases (16/42) and 9% of the controls (4/44) have Hungatella in their microbiome. Relative abundances (%) of the participants who have Hungatella species in their gut microbiome ranges between 0.980% and 0.010%. Thirty-eight percent of the cases (16/42) and 20% of the controls (9/44) have Tyzzerella. Relative abundances (%) of the participants who have Tyzzerella species in their gut microbiome ranges between 2.450% and 0.017%.

The most abundant bacteria at the phylum and genus levels were similar between the groups (Figure 1) and the bacteria identified were consistent with bacteria commonly found in the gut microbiome [38,39]. Importantly, beta diversity analysis identified differences in the overall microbial community composition between patients with BCa and cancer-free controls (Table 4). Additionally, LefSe analysis showed 3 genera that were enriched in fecal samples from BCa patients (Acidaminococcus, Tyzzerella, and Hungatella) and another 10 genera that were separately enriched in controls (Christensenellaceae, UCG-005, Oscillospirales, NK4A14 group, Dialister, Gastranaerophilales, Romboutsia, Coriobacteriales, Anaerofilum, Flavobacterials) (Figure 2). The distribution of the BCa-enriched genera across groups revealed that Acidaminococus was present in 24% of cases (10/42) and 9% of controls (4/44), with abundance ranging between 0.01–1. 85%. Likewise, Hungatella was detected in 38% of cases (16/42) and 9% of controls (4/44), with abundance ranging between 0.01–0.98%. Tyzzerella was found in 38% of cases and 20% of controls, with abundance ranging between 0.017–2.45% (Figure 3).

Since there were no significant differences between cases and controls in their HEI-2015 total and component scores, we further investigated whether the presence of BCa-enriched genera was associated with dietary habits based on HEI-2015 total and component scores (Table 5). We found that Acidaminococus-positive participants had a lower HEI-2015 whole fruit component intake score (*p* = 0.005), whereas Hungatella-positive participants had a lower intake of dairy HEI-2015 component score (*p* = 0.029) and higher intake of total vegetables HEI-2015 component score (*p* = 0.024). Tyzzerella presence was not associated with HEI-2015 dietary intake scores (Table 5).

## 4. Discussion

In this case-control study, we comprehensively analyzed the gut microbiome in BCa patients and cancer-free controls and observed differences in the composition of the gut microbiome between the two groups. Notably, we identified three genera associated with an increased risk of BCa: Acidaminococus, Hungatella, and Tyzzerella. These three taxa were also associated with dietary intake patterns.

Our findings highlight a link between BCa risk and lifestyle factors, including emotional stress, diet, and physical activity, which aligns with prior research [40,41,42]. Notably, we observed that BCa patients had less diversity in their gut microbiomes. This reduction in species richness and evenness is indicative of dysbiosis [43], which can arise from various factors, including dietary habits, antibiotic use, lifestyle choices, and underlying health conditions [43]. This finding also suggests that gut microbiome alpha-diversity may be an effective resource for BCa screening. Our study is consistent with others demonstrating lower gut microbiome alpha-diversity among postmenopausal breast cancer patients, with differences in microbiome composition, independent of estrogen levels [44,45,46,47]. Gut microbiota dysbiosis may be associated with breast cancer risk through both estrogen-dependent and non-estrogen-dependent mechanisms that involve the production of microbial-derived metabolites, immune regulation and effects on DNA [48]. Additionally, microbial metabolites might act as effector molecules, influencing physiological processes and drug metabolism as well as modulating immune responses [49]. Our analysis revealed that BCa cases reported higher levels of emotional stress in the past year and at the age of 25. While the intricate relationship between emotional stress and BCa warrants further investigation, existing studies suggest a connection between microbial diversity, depression, and chronic inflammation. Notably, dysbiosis and reduced microbial diversity can contribute to gut and systemic inflammation, potentially impacting mood and behavior, via production of short-chain fatty acids and neurotransmitter precursors [50,51].

Previous findings from the UK Biobank prospective cohort study indicate that physical activity plays a role in BCa risk, with lower physical activity levels being associated with increased BCa risk [40]. In support of these findings, we observed a significant difference in the levels of total energy expenditure from recreational physical activity between cases and controls (*p* = 0.02). Indeed, regular physical activity, particularly aerobic exercise, is linked to a more diverse and balanced gut microbiome composition [40]. The anti-inflammatory effects of exercise may extend to the gut, helping to maintain a healthy microbial balance [40]. Additionally, physical activity can improve insulin sensitivity and glucose metabolism, creating a favorable environment for beneficial gut bacteria [52]. Individuals engaged in regular physical activity are more often likely to adopt a diverse and balanced diet, which positively influences gut microbial diversity and composition. Conversely, lower physical activity levels are associated with an increased risk of obesity, which itself can alter the gut microbiome and reduce microbial diversity [52]. Building on these concepts, our study identified a significant difference in BMI between BCa cases and controls (*p* = 0.02), emphasizing the importance of lifestyle and weight management in BCa risk.

An examination of the compositional differences in fecal microbiota between BCa cases and controls revealed statistically significant differences that identified enrichment of Acidaminococus, Tyzzerella, and Hungatella within BCa patients’ gut microbiomes. These genera were also associated with dietary habits. Acidaminococus, which is known for its amino acid fermentation capabilities, contributes to the breakdown of proteins and amino acids in the gut, impacting nutrient absorption and gut health [52]. Typically considered commensal and harmless, Acidaminococcus is a diverse genus with multiple species, and little is known about its contributions to disease pathogenesis. For example, emerging data points to Acidaminococcus as the predominant strain in pancreatic cyst fluid [53]. Likewise, the discovery of a novel Cpf1 (Cas12a) CRISPR enzyme from Acidaminococcus and Lachnospiraceae was shown to have efficient genome-editing activity in human cells [54]. Whether bacterium such as Acidaminococcus can use these enzyme systems on neighboring human cells within the alimentary tract against human cells, and possibly initiate (or further contribute) to neoplastic transformation requires further research.

Hungatella is associated with increased trimethylamine N-oxide (TMAO) levels and choline metabolism [55] and is implicated in promoting cell proliferation and angiogenesis in colorectal cancer development [55]. An inverse association between Hungatella and grain consumption suggests a potential dietary influence on its abundance [56]. The Hungatella genera have the highest number of enzymes capable of degrading the glycosaminoglycans (GAGs) that form the protective layer in the colon mucosa. GAGs are an important component of the gut environment, and the breakdown of this layer disrupts the homeostasis in the colon or mucosal structures, as well as the overall balance of the microbiota, which can lead to dysbiosis [57]. Importantly, Hungatella hathewayi has been found to induce DNA methyl transferase activity in colonic epithelial cells which is associated with significant increases in global DNA methylation, including tumor suppressor genes (e.g., CHFR, GATA5, and PAX6), and contributes colorectal tumorigenesis [58]. Our observed differences in the genus of Hungatella between our study groups requires further investigation regarding the role of Hungatella in BCa tumorigenesis.

Tyzzerella, a less-studied genus, has been sporadically linked to inflammatory processes and cardiovascular disease risk [59]. The relative abundance of Tyzzerella is associated with high short-chain fatty acid diets [60]. Interestingly, there is mounting evidence showing that short-chain fatty acids are capable of modulating the efficacy of various anti-cancer treatments (e.g., chemotherapy, immunotherapy, radiotherapy) [61,62,63]. The role of Tyzzerella in the gut and its contribution to BCa risk requires further research.

Demographic variables such as age, race, and family history of BCa, parity status, menopausal status, and menarche age were similar between the two groups, minimizing potential confounding effects on our findings. Even so, our study had several limitations. Firstly, the small sample size of this pilot study requires validation in a larger cohort study. Second, the hospital-based case-control study design cannot delineate a causal relationship between the gut microbiome and BCa, and our study population needs to be expanded to more diverse population-based studies. We also have limited power to conduct granular analyses based on breast cancer molecular subtypes. Future studies should provide a more in-depth examination of the relationship between the gut microbiome biomarkers identified in this study and associated BCa risk. This includes assessment of the fecal metabolome to provide a functional quantitation of their microbial activity and influences on the host and environmental factors. Additionally, research should aim to identify dietary strategies for promoting a healthy gut microbiome that reduces BCa risk.

## 5. Conclusions

Our study identified three genera—Acidaminococus, Tyzzerella, and Hungatella—as potential biomarker candidates associated with the gut microbiomes of BCa patients. Furthermore, our investigation into dietary intakes revealed significant associations between the presence of these genera in the microbiome and specific HEI2015 components, such as vegetables and dairy for Hungatella, and whole fruits for Acidaminococus. These findings suggest a potential link between dietary intakes and the gut microbiome composition, which could have implications for BCa risk and management. Further research is warranted to elucidate the precise mechanisms underlying these associations and their implications for BCa prevention and treatment. Importantly, these findings provide the foundations for empowering individuals to make informed lifestyle choices that may proactively reduce their BCa risk.

## Figures and Tables

**Table 1:** Demographic characteristics of study participants.

Variable	BCa Cases (N = 42)	Controls (N = 44)	*p*-Value
**Demographic Characteristics**			
Age at enrollment, mean (SD), yr	60.3 (11.3)	58.5 (12.6)	0.49
Race, No. (%)			
White	40 (95.2)	41 (93.2)	0.68
Non-White	2 (4.8)	3 (6.8)	
BMI (kg/m^2^) at enrollment, mean (SD) continuous variable	28.1 (6.2)	25.1 (5.6)	**0.02**
Family history of BCa, No. (%)			0.95
Yes	15 (35.7)	16 (36.4)	
No	27 (64.3)	28 (63.6)	
Ever full-term live birth, No. (%)			0.97
Yes	30 (71.4)	28 (71.8)	
No	12 (28.6)	11 (28.2)	
Missing	0	5	
Menopausal status, No. (%)			0.90
Premenopausal	10 (23.8)	11 (25.0)	
Post/peri-menopausal	32 (76.2)	33 (75.0)	
Age at menarche, No. (%)			0.83
≤11 years old	10 (23.8)	9 (20.5)	
12–14 years old	26 (61.9)	30 (68.2)	
≥15 years old	6 (14.3)	5 (11.4)	
Marital Status, No. (%)			0.25
Married/Living as married	27 (64.3)	29 (65.9)	
Divorced/Separated/Widowed	13 (31.0)	9 (20.5)	
Single, never married	2 (4.8)	6 (13.6)	
Employment Status, No. (%)			0.13
Employed/self-employed	20 (47.6)	28 (63.6)	
Unemployed/Disabled/Retired/Homemaker	22 (52.4)	16 (36.4)	

Chi-square tests are used for categorical variables and *t*-tests are used for continuous variables. Fisher’s exact test is used for cells < 5 sample size. Missing categories are excluded from statistical analysis.

**Table 2:** Lifestyle characteristics of study participants.

Variable	BCa Cases (N = 42)	Controls (N = 44)	*p*-Value
**Life Style Characteristics**			
Total dietary energy intake (kcal/day)	1664.9 (752.8)	1521.5 (590.8)	0.33
Total energy expended from recreational physical activity (MET-minutes/week)	2093.9 (1247.1)	2728.4 (1274.4)	**0.02**
Ever Smoked 100 cigarettes in life			0.95
Yes	15 (35.7)	16 (36.4)	
No	27 (64.3)	28 (63.6)	
Alcohol, No. (%)			0.45
Current drinkers	36 (85.7)	34 (77.3)	
Past drinkers	3 (7.1)	7 (15.9)	
Never drinkers	3 (7.1)	3 (6.8)	
Missing			
Sleep Quality Score, No. (%)			0.58
11–14	13 (31.0)	10 (22.7)	
15–16	15 (35.7)	15 (34.1)	
17–18	14 (33.3)	19 (43.2)	
Ever used hormone therapy, No. (%)			0.29
Yes	24 (57.1)	30 (68.2)	
No	18 (42.9)	14 (31.8)	
Regular (at least once a week) Probiotic product use, No. (%)			0.52
Yes	21 (50.0)	19 (43.2)	
No	21 (50.0)	25 (56.8)	
Feeling downhearted and blue in the past year, No. (%)			**0.02**
All/most of the time	8 (19.1)	2 (4.6)	
Some of the time	19 (45.2)	13 (29.6)	
A little of the time	11 (26.2)	19 (43.2)	
None of the time	4 (9.5)	10 (22.7)	
Feeling downhearted and blue around age 25, No. (%)			**0.01**
All/most of the time	2 (5.0)	4 (9.1)	
Some of the time	9 (22.5)	12 (27.3)	
A little of the time	17 (42.5)	15 (34.1)	
None of the time	12 (30.0)	13 (27.3)	
Don’t know	2	0	

Chi-square tests are used for categorical variables and *t*-tests are used for continuous variables. Fisher’s exact test is used for cells < 5 sample size. Missing categories are excluded from statistical analysis. Sleep quality score is a composite score calculated from 6 questions regarding sleep regularity, satisfaction, alertness, timing, efficiency, and duration with 3 Likert scale responses: “Rarely/Never (1), Sometimes (2), Usually always (3)”. The score range is 6–18. Probiotic products include tablets, yogurt, drinks, and other products.

**Table 3:** Alpha-diversity analysis between BCa cases and healthy control group participants.

Alpha Diversity	Cases	Controls	*p*-Value (Unadjusted) ANOVA	*p*-Value (Adjusted BMI) ANOVA	Wilcoxon Rank Sum Test with Continuity Correction *p*-Value
**Phylum Level**					
Observed	198.57 (52.14)	228.73 (68.94)	**0.025**	**0.025**	**0.045**
Shannon	3.91 (0.40)	4.13 (0.42)	**0.013**	**0.012**	**0.013**
Inverse Simpson	25.97 (11.18)	34.22 (15.47)	**0.006**	**0.005**	**0.007**
Pielou	0.74 (0.06)	0.77 (0.04)	**0.030**	**0.028**	**0.009**

Alpha-diversity analysis between BC and healthy control group participants. All alpha-diversity indices were analyzed using the ANOVA and Wilcoxon rank-sum test. The richness of the fecal microbiota of case patients, adjusted BMI, compared with control patients, had statistically lower alpha diversity with Shannon index (*p* = 0.012), Observed (*p* = 0.025), Inverse Simpson (*p* = 0.005), Pielou (*p* = 0.028).

**Table 4:** Beta Diversity- Pairwise Permanova Analysis between BCa cases and healthy control group participants.

Case-Control	Sample Size	Permutations	pseudo_F	*p*-Value	q-Value
Jaccard-significance	86	999	1.261412	0.04	0.04
Bray-Curtis-significance	86	999	1.121184	0.23	0.23

The statistical significance of beta-diversity was measured using permutational multivariate analysis of variance (PERMANOVA). The statistical significance of fecal microbiota compositions observed in the Jaccard analysis is (*p* = 0.04) for beta diversity between the two groups. The statistical significance of fecal microbiota compositions observed in the Bray-Curtis analysis is (0.23) for beta diversity between the two groups.

**Table 5:** Distribution of HEI index by selected bacteria presence/absence status.

Healthy Eating Index 2015	Min-Max Score	Case-Control Status			Acidaminococus			Hungatella			Tyzzerella		
		Cases (n = 39)	Controls (n = 44)	*p* Value	Positive (n = 14)	Negative (n = 69)	*p* Value	Positive (n = 18)	Negative (n = 65)	*p* Value	Positive (n = 24)	Negative (n = 59)	*p* Value
**Adequacy**													
Total vegetable score	0–5	5.00 (0.60)	4.90 (1.13)	0.25	5.00 (1.40)	5.00 (0.59)	0.989	5.00 (0.00)	4.88 (1.20)	**0.024**	5.00 (0.63)	5.00 (1.19)	0.282
Greens and beans score	0–5	5.00 (0.00)	5.00 (0.00)	0.83	5.00 (3.05)	5.00 (0)	0.055	5.00 (0.00)	5.00 (0.00)	0.73	5.00 (0.00)	5.00 (0.00)	0.941
Total fruits score	0–5	5.00 (2.61)	5.00 (0.59)	0.23	5.00 (4.10)	5.00 (1.35)	0.06	5.00 (1.35)	5.00 (2.01)	0.749	5.00 (1.05)	5.00 (2.01)	0.417
Whole fruits score	0–5	5.00 (1.40)	5.00 (0.00)	0.10	4.50 (3.31)	5.00 (0.00)	**0.005**	5.00 (0.00)	5.00 (0.00)	0.573	5.00 (0.55)	5.00 (0.00)	0.795
Whole grains score	0–10	2.68 (2.32)	3.45 (2.96)	0.25	2.76 (3.89)	3.15 (2.41)	0.507	2.60 (2.33)	3.15 (2.43)	0.973	2.31 (3.12)	3.15 (2.48)	0.563
Dairy score	0–10	5.88 (4.52)	6.97 (4.70)	0.27	5.73 (5.49)	6.60 (4.25)	0.22	4.99 (4.22)	6.60 (4.31)	**0.029**	5.39 (4.99)	7.01 (3.72)	0.084
Total protein food score	0–5	5.00 (0.72)	5.00 (0.00)	0.29	5.00 (1.02)	5.00 (0.00)	0.511	5.00 (1.02)	5.00 (0.00)	0.207	5.00 (0.33)	5.00 (0.00)	0.702
Seafood and plant proteins score	0–5	5.00 (0.21)	5.00 (0.00)	0.35	5.00 (0.15)	5.00 (0.00)	0.679	5.00 (0.93)	5.00 (0.00)	0.1	5.00 (0.55)	5.00 (0.00)	0.58
Fatty acids score	0–10	5.5 (4.90)	5.22 (7.21)	0.65	4.52 (5.80)	5.26 (4.41)	0.591	6.20 (6.68)	5.06 (4.65)	0.694	5.24 (5.64)	5.24 (4.51)	0.259
**Moderation**													
Sodium score	0–10	5.25 (2.44)	5.25 (2.12)	0.99	4.50 (2.18)	5.40 (2.26)	0.18	5.76 (2.69)	5.11 (2.13)	0.28	5.49 (2.06)	5.15 (2.34)	0.55
Refined grains	0–10	9.96 (1.56)	10.00 (1.25)	0.28	10.00 (2.49)	10.00 (1.26)	0.178	9.56 (1.60)	10.00 (1.18)	0.326	10.00 (0.54)	9.96 (1.56)	0.126
Saturated fat	0–10	6.27 (3.34)	6.32 (5.44)	0.87	6.44 (4.61)	6.23 (4.84)	0.883	6.88 (4.91)	5.85 (4.26)	0.232	10.00 (4.07)	5.55 (4.48)	0.116
Added sugar	0–10	9.19 (2.41)	9.52 (2.36)	0.68	8.95 (2.93)	9.54 (2.29)	0.243	9.14 (2.93)	9.50 (2.37)	0.502	9.49 (2.02)	9.30 (2.44)	0.946
**Total HEI-2015 Score**	0–100	70.27 (13.08)	75.77 (15.37)	0.17	68.80 (32.20)	72.73 (14.39)	0.45	70.55 (10.16)	72.73 (15.19)	0.48	75.11 (13.50)	71.28 (15.19)	0.14

Sodium score is normally distributed and is expressed as mean (standard deviation), all the other variables are not normally distributed and are expressed as median (IQR). Three breast cancer patients did not complete dietary intake data, therefore, they were not included in the diet-related analyses. *p*-values for total HEI-2015 score were age and BMI-adjusted.

## Data Availability

In accordance with local privacy regulations, the requested data is available upon formal request and approval.

## Supplementary Materials

The following supporting information can be downloaded at: https://www.mdpi.com/article/10.3390/nu15214628/s1, Figure S1: Sample Size Flow Chart.

## References

[1] Sung H., Ferlay J., Siegel R.L., Laversanne M., Soerjomataram I., Jemal A., Bray F. (2021). Global Cancer Statistics 2020: GLOBOCAN Estimates of Incidence and Mortality Worldwide for 36 Cancers in 185 Countries. CA Cancer J. Clin..

[2] Giaquinto A.N., Sung H., Miller K.D., Kramer J.L., Newman L.A., Minihan A., Jemal A., Siegel R.L. (2022). Breast Cancer Statistics, 2022. CA Cancer J. Clin..

[3] Britt K.L., Cuzick J., Phillips K.A. (2020). Key steps for effective breast cancer prevention. Nat. Rev. Cancer.

[4] Uhomoibhi T.O., Okobi T.J., Okobi O.E., Koko J.O., Uhomoibhi O., Igbinosun O.E., Ehibor U.D., Boms M.G., Abdulgaffar R.A., Hammed B.L. (2022). High-Fat Diet as a Risk Factor for Breast Cancer: A Meta-Analysis. Cureus.

[5] Kazemi A., Barati-Boldaji R., Soltani S., Mohammadipoor N., Esmaeilinezhad Z., Clark C.C.T., Babajafari S., Akbarzadeh M. (2021). Intake of Various Food Groups and Risk of Breast Cancer: A Systematic Review and Dose-Response Meta-Analysis of Prospective Studies. Adv. Nutr..

[6] Chen X., Wang Q., Zhang Y., Xie Q., Tan X. (2019). Physical Activity and Risk of Breast Cancer: A Meta-Analysis of 38 Cohort Studies in 45 Study Reports. Value Health.

[7] Sun Q., Xie W., Wang Y., Chong F., Song M., Li T., Xu L., Song C. (2020). Alcohol Consumption by Beverage Type and Risk of Breast Cancer: A Dose-Response Meta-Analysis of Prospective Cohort Studies. Alcohol Alcohol..

[8] He Y., Si Y., Li X., Hong J., Yu C., He N. (2022). The relationship between tobacco and breast cancer incidence: A systematic review and meta-analysis of observational studies. Front. Oncol..

[9] Sarhangi N., Hajjari S., Heydari S.F., Ganjizadeh M., Rouhollah F., Hasanzad M. (2022). Breast cancer in the era of precision medicine. Mol. Biol. Rep..

[10] Eslami S.Z., Majidzadeh A.K., Halvaei S., Babapirali F., Esmaeili R. (2020). Microbiome and Breast Cancer: New Role for an Ancient Population. Front. Oncol..

[11] Shreiner A.B., Kao J.Y., Young V.B. (2015). The gut microbiome in health and in disease. Curr. Opin. Gastroenterol..

[12] Bodai B.I., Nakata T.E. (2020). Breast Cancer: Lifestyle, the Human Gut Microbiota/Microbiome, and Survivorship. Perm. J..

[13] Wu A.H., Tseng C., Vigen C., Yu Y., Cozen W., Garcia A.A., Spicer D. (2020). Gut microbiome associations with breast cancer risk factors and tumor characteristics: A pilot study. Breast Cancer Res. Treat..

[14] Jaye K., Chang D., Li C.G., Bhuyan D.J. (2022). Gut Metabolites and Breast Cancer: The Continuum of Dysbiosis, Breast Cancer Risk, and Potential Breast Cancer Therapy. Int. J. Mol. Sci..

[15] Zheng D., Liwinski T., Elinav E. (2020). Interaction between microbiota and immunity in health and disease. Cell Res..

[16] Belkaid Y., Hand T.W. (2014). Role of the microbiota in immunity and inflammation. Cell.

[17] Feng T.Y., Azar F.N., Dreger S.A., Rosean C.B., McGinty M.T., Putelo A.M., Kolli S.H., Carey M.A., Greenfield S., Fowler W.J. (2022). Reciprocal Interactions Between the Gut Microbiome and Mammary Tissue Mast Cells Promote Metastatic Dissemination of HR+ Breast Tumors. Cancer Immunol. Res..

[18] Laborda-Illanes A., Sanchez-Alcoholado L., Boutriq S., Plaza-Andrades I., Peralta-Linero J., Alba E., Gonzalez-Gonzalez A., Queipo-Ortuno M.I. (2021). A New Paradigm in the Relationship between Melatonin and Breast Cancer: Gut Microbiota Identified as a Potential Regulatory Agent. Cancers.

[19] Fernandez M.F., Reina-Perez I., Astorga J.M., Rodriguez-Carrillo A., Plaza-Diaz J., Fontana L. (2018). Breast Cancer and Its Relationship with the Microbiota. Int. J. Environ. Res. Public Health.

[20] Singh R.K., Chang H.W., Yan D., Lee K.M., Ucmak D., Wong K., Abrouk M., Farahnik B., Nakamura M., Zhu T.H. (2017). Influence of diet on the gut microbiome and implications for human health. J. Transl. Med..

[21] Mahmood R., Voisin A., Olof H., Khorasaniha R., Lawal S.A., Armstrong H.K. (2023). Host Microbiomes Influence the Effects of Diet on Inflammation and Cancer. Cancers.

[22] (2020). Diet History Questionnaire.

[23] Reedy J., Lerman J.L., Krebs-Smith S.M., Kirkpatrick S.I., Pannucci T.E., Wilson M.M., Subar A.F., Kahle L.L., Tooze J.A. (2018). Evaluation of the Healthy Eating Index-2015. J. Acad. Nutr. Diet..

[24] Ware J., Kosinski M., Keller S.D. (1996). A 12-Item Short-Form Health Survey: Construction of scales and preliminary tests of reliability and validity. Med. Care.

[25] Caporaso J.G., Lauber C.L., Walters W.A., Berg-Lyons D., Lozupone C.A., Turnbaugh P.J., Fierer N., Knight R. (2011). Global patterns of 16S rRNA diversity at a depth of millions of sequences per sample. Proc. Natl. Acad. Sci. USA.

[26] Callahan B.J., McMurdie P.J., Rosen M.J., Han A.W., Johnson A.J., Holmes S.P. (2016). DADA2: High-resolution sample inference from Illumina amplicon data. Nat. Methods.

[27] Quast C., Pruesse E., Yilmaz P., Gerken J., Schweer T., Yarza P., Peplies J., Glockner F.O. (2013). The SILVA ribosomal RNA gene database project: Improved data processing and web-based tools. Nucleic Acids Res..

[28] Price M.N., Dehal P.S., Arkin A.P. (2010). FastTree 2--approximately maximum-likelihood trees for large alignments. PLoS ONE.

[29] McMurdie P.J., Holmes S. (2013). phyloseq: An R package for reproducible interactive analysis and graphics of microbiome census data. PLoS ONE.

[30] Whittaker R.H. (1960). Vegetation of the Siskiyou Mountains, Oregon and California. Ecol. Monogr..

[31] Shannon C.E. (1948). A mathematical theory of communication. Bell Syst. Technol. J..

[32] Simpson E.H. (1949). Measurement of Diversity. Nature.

[33] Gotelli N.J., Colwell R.K. (2001). Quantifying biodiversity: Procedures and pitfalls in the measurement and comparison of species richness. Ecol. Lett..

[34] Pielou E.C. (1966). The measurement of diversity in different types of biological collections. J. Theor. Biol..

[35] Schroeder P.J., Jenkins D.G. (2018). How robust are popular beta diversity indices to sampling error?. Ecosphere.

[36] Legendre P., De Cáceres M. (2013). Beta diversity as the variance of community data: Dissimilarity coefficients and partitioning. Ecol. Lett..

[37] Segata N., Izard J., Waldron L., Gevers D., Miropolsky L., Garrett W.S., Huttenhower C. (2011). Metagenomic biomarker discovery and explanation. Genome Biol..

[38] Arumugam M., Raes J., Pelletier E., Le Paslier D., Yamada T., Mende D.R., Fernandes G.R., Tap J., Bruls T., Batto J.M. (2011). Enterotypes of the human gut microbiome. Nature.

[39] Rinninella E., Raoul P., Cintoni M., Franceschi F., Miggiano G.A.D., Gasbarrini A., Mele M.C. (2019). What is the Healthy Gut Microbiota Composition? A Changing Ecosystem across Age, Environment, Diet, and Diseases. Microorganisms.

[40] Guo W., Fensom G.K., Reeves G.K., Key T.J. (2020). Physical activity and breast cancer risk: Results from the UK Biobank prospective cohort. Br. J. Cancer.

[41] Heath A.K., Muller D.C., van den Brandt P.A., Papadimitriou N., Critselis E., Gunter M., Vineis P., Weiderpass E., Fagherazzi G., Boeing H. (2020). Nutrient-wide association study of 92 foods and nutrients and breast cancer risk. Breast Cancer Res..

[42] Zhu G.L., Xu C., Yang K.B., Tang S.Q., Tang L.L., Chen L., Li W.F., Mao Y.P., Ma J. (2022). Causal relationship between genetically predicted depression and cancer risk: A two-sample bi-directional mendelian randomization. BMC Cancer.

[43] Carding S., Verbeke K., Vipond D.T., Corfe B.M., Owen L.J. (2015). Dysbiosis of the gut microbiota in disease. Microb. Ecol. Health Dis..

[44] Bobin-Dubigeon C., Luu H.T., Leuillet S., Lavergne S.N., Carton T., Le Vacon F., Michel C., Nazih H., Bard J.M. (2021). Faecal Microbiota Composition Varies between Patients with Breast Cancer and Healthy Women: A Comparative Case-Control Study. Nutrients.

[45] Byrd D.A., Vogtmann E., Wu Z., Han Y., Wan Y., Clegg-Lamptey J.N., Yarney J., Wiafe-Addai B., Wiafe S., Awuah B. (2021). Associations of fecal microbial profiles with breast cancer and nonmalignant breast disease in the Ghana Breast Health Study. Int. J. Cancer.

[46] Hou M.F., Ou-Yang F., Li C.L., Chen F.M., Chuang C.H., Kan J.Y., Wu C.C., Shih S.L., Shiau J.P., Kao L.C. (2021). Comprehensive profiles and diagnostic value of menopausal-specific gut microbiota in premenopausal breast cancer. Exp. Mol. Med..

[47] Goedert J.J., Jones G., Hua X., Xu X., Yu G., Flores R., Falk R.T., Gail M.H., Shi J., Ravel J. (2015). Investigation of the association between the fecal microbiota and breast cancer in postmenopausal women: A population-based case-control pilot study. J. Natl. Cancer Inst..

[48] Ruo S.W., Alkayyali T., Win M., Tara A., Joseph C., Kannan A., Srivastava K., Ochuba O., Sandhu J.K., Went T.R. (2021). Role of Gut Microbiota Dysbiosis in Breast Cancer and Novel Approaches in Prevention, Diagnosis, and Treatment. Cureus.

[49] Parida S., Sharma D. (2020). Microbial Alterations and Risk Factors of Breast Cancer: Connections and Mechanistic Insights. Cells.

[50] Kelly J.R., Kennedy P.J., Cryan J.F., Dinan T.G., Clarke G., Hyland N.P. (2015). Breaking down the barriers: The gut microbiome, intestinal permeability and stress-related psychiatric disorders. Front. Cell. Neurosci..

[51] Han K.M., Ham B.J. (2021). How Inflammation Affects the Brain in Depression: A Review of Functional and Structural MRI Studies. J. Clin. Neurol..

[52] Asfaw M.S., Dagne W.K. (2022). Physical activity can improve diabetes patients’ glucose control; A systematic review and meta-analysis. Heliyon.

[53] Li S., Fuhler G.M., Bn N., Jose T., Bruno M.J., Peppelenbosch M.P., Konstantinov S.R. (2017). Pancreatic cyst fluid harbors a unique microbiome. Microbiome.

[54] Zetsche B., Gootenberg J.S., Abudayyeh O.O., Slaymaker I.M., Makarova K.S., Essletzbichler P., Volz S.E., Joung J., van der Oost J., Regev A. (2015). Cpf1 is a single RNA-guided endonuclease of a class 2 CRISPR-Cas system. Cell.

[55] al-Waiz M., Mikov M., Mitchell S.C., Smith R.L. (1992). The exogenous origin of trimethylamine in the mouse. Metabolism.

[56] Genoni A., Christophersen C.T., Lo J., Coghlan M., Boyce M.C., Bird A.R., Lyons-Wall P., Devine A. (2020). Long-term Paleolithic diet is associated with lower resistant starch intake, different gut microbiota composition and increased serum TMAO concentrations. Eur. J. Nutr..

[57] Rawat P.S., Li Y., Zhang W., Meng X., Liu W. (2022). Hungatella hathewayi, an Efficient Glycosaminoglycan-Degrading Firmicutes from Human Gut and Its Chondroitin ABC Exolyase with High Activity and Broad Substrate Specificity. Appl. Environ. Microbiol..

[58] Xia X., Wu W.K.K., Wong S.H., Liu D., Kwong T.N.Y., Nakatsu G., Yan P.S., Chuang Y.-M., Chan M.W.-Y., Coker O.O. (2020). Bacteria pathogens drive host colonic epithelial cell promoter hypermethylation of tumor suppressor genes in colorectal cancer. Microbiome.

[59] Kelly T.N., Bazzano L.A., Ajami N.J., He H., Zhao J., Petrosino J.F., Correa A., He J. (2016). Gut Microbiome Associates With Lifetime Cardiovascular Disease Risk Profile Among Bogalusa Heart Study Participants. Circ. Res..

[60] Xu A.A., Kennedy L.K., Hoffman K., White D.L., Kanwal F., El-Serag H.B., Petrosino J.F., Jiao L. (2022). Dietary Fatty Acid Intake and the Colonic Gut Microbiota in Humans. Nutrients.

[61] Al-Qadami G.H., Secombe K.R., Subramaniam C.B., Wardill H.R., Bowen J.M. (2022). Gut Microbiota-Derived Short-Chain Fatty Acids: Impact on Cancer Treatment Response and Toxicities. Microorganisms.

[62] He Y., Fu L., Li Y., Wang W., Gong M., Zhang J., Dong X., Huang J., Wang Q., Mackay C.R. (2021). Gut microbial metabolites facilitate anticancer therapy efficacy by modulating cytotoxic CD8(+) T cell immunity. Cell Metab..

[63] Rangan P., Mondino A. (2022). Microbial short-chain fatty acids: A strategy to tune adoptive T cell therapy. J. Immunother. Cancer.

