# Supplementary figures and images for: Association between Gut Microbiota and Breast Cancer: Diet as a Potential Modulating Factor
Source: Nutrients. 2023 Oct 31;15(21):4628. doi: 10.3390/nu15214628 (PMC10649662; doi:10.3390/nu15214628)

**Supplemental Figure S1. Sample Size Flow Chart**

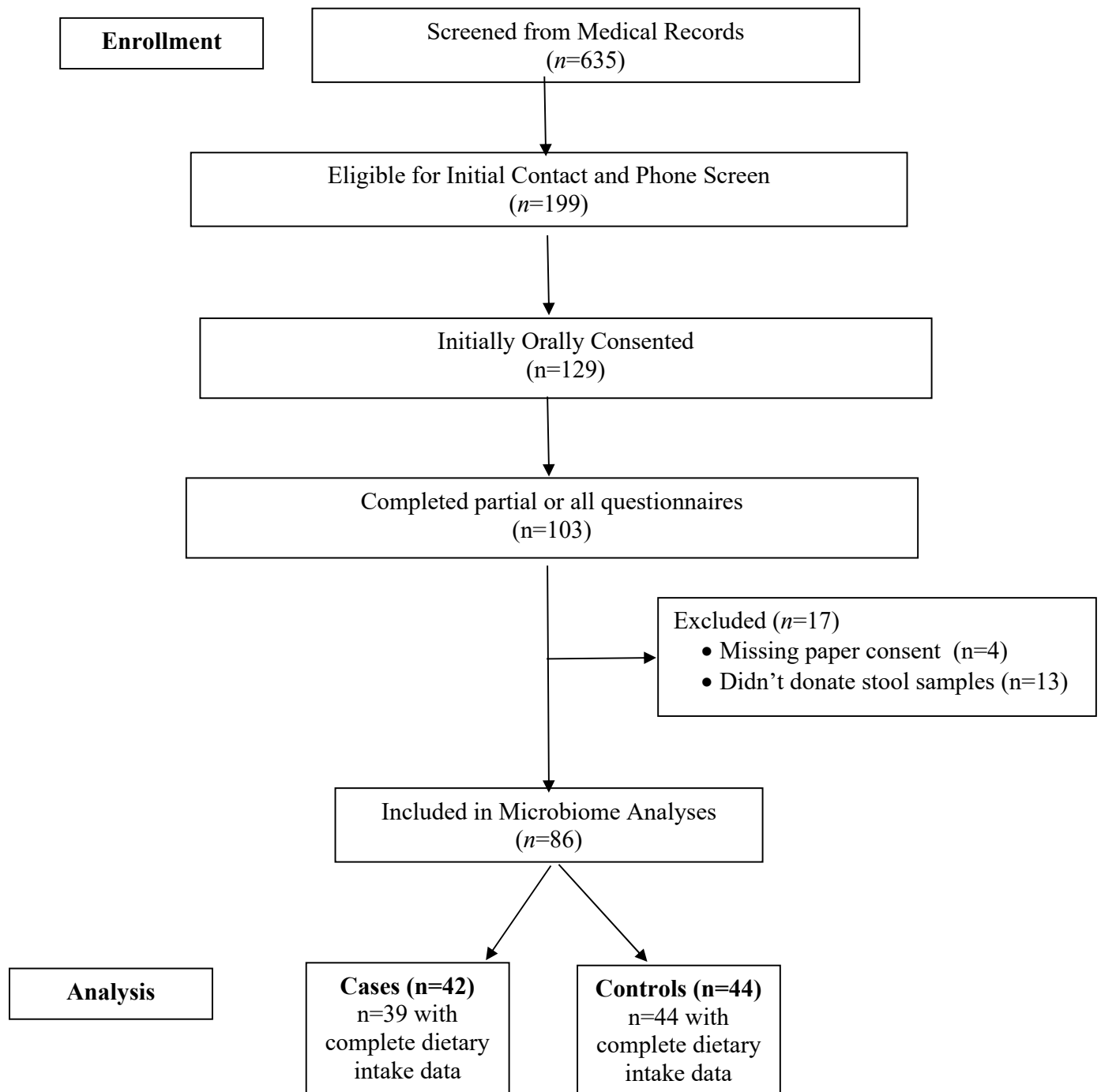

Supplement: Supplementary file 1 [file nutrients-15-04628-s001.zip › nutrients-2676281-supplementary.pdf]
